# Supplementary figures and images for: The Effects of Vitamin D on the Expression of IL-33 and Its Receptor ST2 in Skin Cells; Potential Implication for Psoriasis
Source: Int J Mol Sci. 2021 Nov 29;22(23):12907. doi: 10.3390/ijms222312907 (PMC8657669; doi:10.3390/ijms222312907)

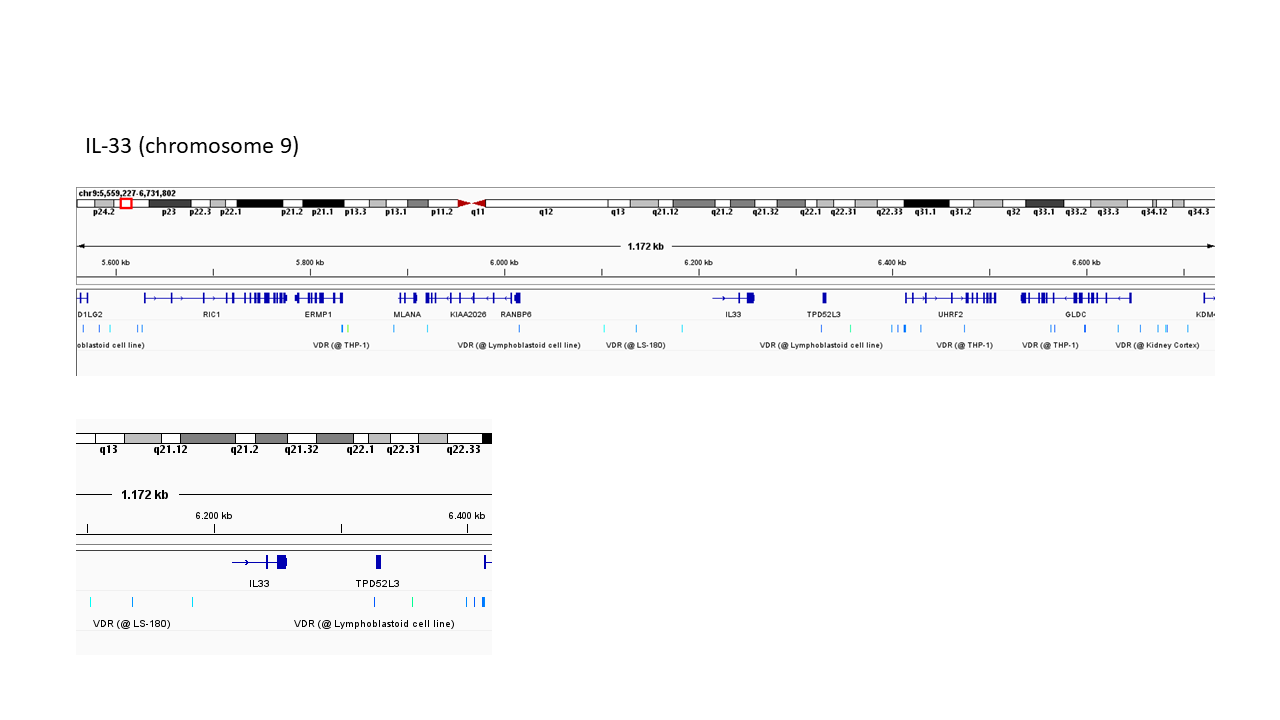

Supplement: Supplementary file 1 [file ijms-22-12907-s001.zip › Supp. Figure S1.tif]

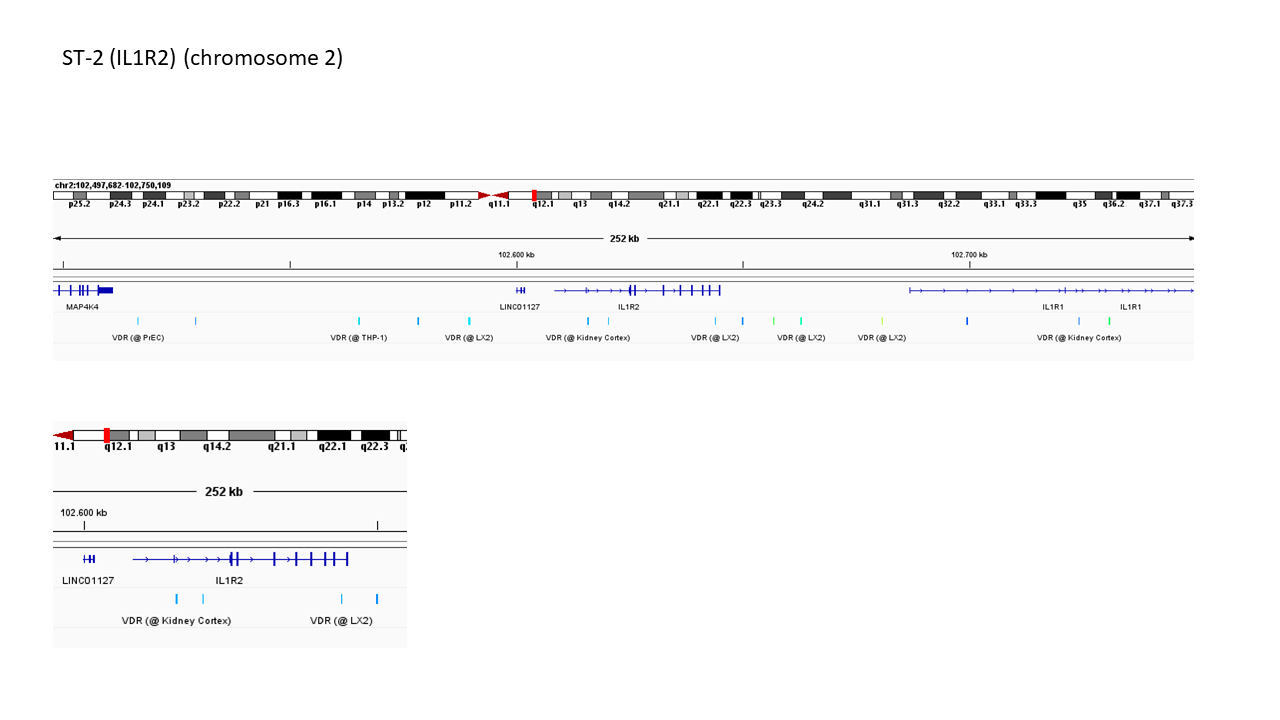

Supplement: Supplementary file 1 [file ijms-22-12907-s001.zip › Supp. Figure S2.tif]
